# The neural correlation of emotion recognition ability and depressive symptoms–evidence from the HCP database

**DOI:** 10.3389/fpsyt.2022.1090369

**Published:** 2023-01-25

**Authors:** Ze Yuan, Xiao Lin, Peng Li, Yu-Jun Gao, Kai Yuan, Wei Yan, Yu-Xin Zhang, Lin Liu, Xi-Mei Zhu, Yi-Jing Zhang, Yan-Ping Bao, Su-Hua Chang, Lin Lu, Le Shi

**Affiliations:** ^1^Savaid Medical School, University of Chinese Academy of Sciences, Beijing, China; ^2^Chinese Academy of Medical Sciences Research Unit (No. 2018RU006), NHC Key Laboratory of Mental Health (Peking University), National Clinical Research Center for Mental Disorders (Peking University Sixth Hospital), Peking University Sixth Hospital, Peking University Institute of Mental Health, Peking University, Beijing, China; ^3^Department of Psychiatry, Renmin Hospital of Wuhan University, Wuhan, China; ^4^Peking-Tsinghua Centre for Life Sciences and PKU-IDG/McGovern Institute for Brain Research, Peking University, Beijing, China; ^5^Beijing Key Laboratory of Drug Dependence, National Institute on Drug Dependence, Peking University, Beijing, China; ^6^School of Public Health, Peking University, Beijing, China

**Keywords:** depressive symptoms, emotional recognition ability, magnetic resonance imaging, functional connectivity, machine learning

## Abstract

**Introduction:**

Negative bias of emotional face is the core feature of depression, but its underlying neurobiological mechanism is still unclear. The neuroimaging findings of negative emotional recognition and depressive symptoms are inconsistent.

**Methods:**

The neural association between depressive symptoms and negative emotional bias were analyzed by measuring the associations between resting state functional connectivity (FC), brain structures, negative emotional bias, and depressive problems. Then, we performed a mediation analysis to assess the potential overlapping neuroimaging mechanisms.

**Results:**

We found a negative correlation between depressive symptoms and emotional recognition. Secondly, the structure and function of the inferior and lateral orbitofrontal gyrus are related to depressive symptoms and emotional recognition. Thirdly, the thickness of the inferior orbitofrontal cortex and the FC between the inferior orbitofrontal gyrus and fusiform gyrus, precuneate and cingulate gyrus mediated and even predicted the interaction between emotion recognition and depressive symptoms. Finally, in response to a negative stimulus, the activation of the frontal pole and precuneus lobe associated with the inferior orbitofrontal gyrus was higher in participants with depressive symptoms.

**Conclusion:**

The core brain regions centered on the inferior orbitofrontal cortex such as middle temporal gyrus, precuneus lobe, frontal pole, insula and cingulate gyrus are the potential neuroimaging basis for the interaction between depressive symptoms and emotional recognition.

## 1. Introduction

Depression is one of the most common mental illnesses and the second leading cause of disability and incapacity worldwide (1). Although neurobiological research has been carried out for more than 60 years, its pathophysiological understanding is still limited (2–4). Negative emotional recognition bias and reduced pleasure experience are two core features and symptoms of depression. Patients with depression paid more attention to negative emotions and tended to classify fuzzy/neutral faces as negative. Patients with depression showed a reduced tendency to recognize positive emotions, such as recognizing joyful faces as neutral faces, resulting in a lack of happiness and identity in their previous motivational activities (5) and motivation (6). Therefore, it is critical to study the relationship and overlapped neural mechanisms between depressive symptoms and emotional recognition bias to prevent and treat depression.

Many studies have shown that in major depression disease (MDD) patients, the dominant facial emotion recognition is destroyed (7) and it is related to the appearance of depression symptoms which are the basis and process of depression (8). Individuals with depression have also been found to have recognition bias, but the evidence of this negative bias was inconsistent (9). Previous small sample functional neuroimaging studies showed that patients with depression have abnormal emotional recognition in emotion-related brain regions (10–15), consisting of the amygdala, medial forehead, insular, anterior cingulate gyrus, nucleus accumbens, and orbitofrontal gyrus (16–18). Neuroimaging studies found that the activation of the prefrontal cortex and anterior cingulate gyrus during emotional recognition was a key feature of major depression disease (19). Resting-state fMRI also found that depressed patients had enhanced FC (functional connectivity) between the parahippocampal gyrus, temporal pole and infratemporal gyrus, and the medial orbitofrontal cortex (20, 21). Results of Granger causality analysis suggest that these areas have a solid driving effect on the medial orbitofrontal cortex (reward-related regions), so it may help to clarify that happiness is reduced in depression (22). The increased FC strength between the temporal cortex and precuneus in patients with depression may be related to the representation of self-consciousness (23), which makes patients with depression a more negative biased (22). For sMRI evidence, in a recent large-scale study of adolescent brain cognitive development datasets, depression was associated with increased volume of the orbital frontal cortex, temporal cortex, and medial frontal cortex (24). There is also some evidence of structural changes in the lateral orbitofrontal cortex in patients with depression (24, 25). Gray matter volume has increased in the posterolateral orbitofrontal cortex and the anterior cingulate cortex (26). Meta-analysis showed that the volume of frontal lobe regions, especially the anterior cingulate gyrus and orbitofrontal cortex, increased in patients with depression (27). It can be seen from the above evidence that emotion recognition bias in depressed individuals is related to orbitofrontal cortex but whether emotion recognition bias is related to depressed symptoms and the impact process is still unclear. Therefore, this study aims to clarify the relationship between depressive symptoms and emotional recognition bias and the role of the orbitofrontal cortex in the interaction between them.

Through the large sample of the international open database HCP (Human Connectome Project), this study explores the relationship between depressive symptoms, emotional recognition, the underlying overlapped neural mechanism, and the common intermediary factors. The main contents of this paper are as follows: (1) using the demographic data, psychological scale, and emotional recognition task test, we analyzed the demographic and behavioral data to explore the relationship between emotion recognition ability and depressive symptoms and whether there are common intermediary factors; (2) the brain regions and FCs related to emotional recognition ability and depressive symptoms are explored; (3) we explored the intermediary relationship between emotion recognition and depressive symptoms, and verified the hypothesis that the cortex near the orbitofrontal gyrus is more related to emotional recognition under depressive symptoms.

## 2. Materials and methods

### 2.1. Participants

To explore the relationship between emotional recognition bias and depressive symptoms, we analyzed phenotype data and imaging data from the Human Connectome Project (HCP) database (March 2017 public data release) from the Washington University-University of Minnesota (WU-Minn HCP) Consortium. We selected the participants with general demographic data, completed the emotional recognition task, ASR (Achenbach Adult Self-Report) Scale, and completed the structural state, resting state and task state MRI scanning for this analysis.

### 2.2. Measurement

The behavioral data analyzed in this study included the ASR depression scale (level 1) and the NIH toolbox emotional task test. ASR scale is a self-assessment tool compiled by Achenbach et al. in 1997 (28, 29). This psychological scale based on DSM-IV (Diagnostic and Statistical Manual of Mental Disorders-IV) was used to evaluate the depressive symptoms of participants in HCP. Depression syndrome scale score, age, and sex corrected depression syndrome scale score, depression scale DSM score, age, and sex corrected depression scale DSM score is also measured to evaluate the level of depressive symptoms. The higher the score, the higher the level of depressive symptoms of the participants. ASR scale is a continuous variable, so it can be used to conduct correlation analysis with the performance of emotion recognition tasks and conduct analysis on the mediating effect of FCs. The emotional NIH toolbox contains only self-reported measurements of emotional recognition functions. Therefore, we used the Pennsylvania Emotion Recognition Test to obtain behavioral measurements of emotional recognition (30–32). In this study, we used the accuracy of the emotion recognition task as the standard of emotion recognition, and we used the completion time of the emotion recognition task to assist in the prediction of depressive symptoms and intermediary effect calculation.

Besides, this study also extracted general demographic data, including gender, age, race, annual family income, years of education, left and right-handedness, body mass index (BMI), blood pressure, marijuana addiction diagnosis history, alcohol addiction diagnosis history, tobacco addiction diagnosis history and addiction drug urine test results. In addition, to explore the effect of psychological factors on the relationship between emotion recognition and depressive symptoms, we included several psychological factors, including personality factors, cognitive factors, stress level, indicators of working memory tasks and social tasks, etc. Some participants had experienced one of the nine depression diagnostic items in the DSM-IV and were counted as participants with depressive symptoms.

All participants performed MRI image acquisition by the HCP item group on a custom Siemens 3T “Connectome Skyra” machine at Washington University in St. Louis, using a standard 32-channel Siemens receiver head coil and a transmission coil customized for a smaller space.

### 2.3. Statistical analysis of data

#### 2.3.1. Statistical analysis of demographic and behavioral data

According to the data in HCP, the general demographic data, the indicators of depressive symptoms, emotional recognition and MRI data were used for analysis in this study. After excluding the missing values, 995 participants were included, and 260 of 995 people had experienced depressive symptoms. With or without depressive symptoms are more suitable for predictive analysis because they are binary values. The data were analyzed using SPSS 20.0 statistical software, including correlation and intermediary analysis. The covariates included sex, race, annual family income, diagnosis history of marijuana addiction, diagnosis history of alcohol addiction, diagnosis history of tobacco addiction, urine test results of addiction to drugs, blood pressure, and anxiety syndrome score of ASR-DSM anxiety scale. Pearson partial correlation analysis was used to explore the relationship between indicators of depressive symptoms and emotional recognition ability and the psychological factors contained in the HCP database. When *p* < 0.05, it is considered that there is a statistically significant correlation. Then, we used simple intermediary analysis to analyze whether the psychological factors (mediating variable M) mediate the relationship between depressive symptoms (independent variable X) and emotional recognition (dependent variable Y) or between emotion recognition (independent variable X) and depressive symptoms (dependent variable Y). In this study, through the PROCESS plug-in of R (33), model 4 of the Bootstrap method is used for simple intermediary effect tests.

#### 2.3.2. Statistical analysis of structural MRI data

Based on the sMRI data obtained from HCP, which has been preprocessed by using the HCP pipeline, a linear regression model was constructed to calculate whether each brain region structure was related to behavioral data (depressive symptoms or emotional recognition ability), including the cortical surface area and thickness of 68 brain regions and the volume of 44 subcortical structures (for detailed information see Supplementary material). Based on the covariates of correlation analysis, total gray matter volume and white matter volume were added as covariables, and multiple comparison correction was carried out using the False Discovery Rate (FDR) method. After FDR correction, *p* < 0.05 is suggested that the brain structure is related to behavioral performance.

#### 2.3.3. Statistical analysis of resting state fMRI and task-related fMRI

Based on fMRI data after HCP preprocessing, this study used MATLAB R2016a software and python 3.6 to deal with resting fMRI and further analyses the ALFF (amplitude of low-frequency fluctuation), fALFF (fractional amplitude of low-frequency fluctuation), ReHo (Regional Homogeneity), VMHC (voxel-mirrored homotopic connectivity), and FC. BrainNetViewer toolkit was used to visualize the results. CONN (FC toolbox) was used to analyze task-related fMRI data. The primary analysis steps include the following:

(1) Data collation: This step only includes participants with two resting fMRI data, excludes those who are missing, and then averages the two resting fMRI data of each participant.

(2) The brain region template selection: The international brain region template Shen template (34) was selected in this study.

(3) ReHo lies in describing the similarity of the time series of a given voxel to the time series of its nearest neighbors, so ReHo is used to describe consistency within a region. ALFF and fALFF are used to reveal BOLD (blood oxygen level dependence) signal strength of spontaneous regional activity and reflect the spontaneous activity of the nerve. VMHC approach aims to reflect the difference in FC between brain hemispheres. Dpabi (Data Processing and Analysis of Brain Imaging) was used to calculate the ALFF, fALFF, ReHo, and VMHC values with and without depressive symptoms and for participants with low (wrong more than once on all emotion recognition tests) and high (all right or wrong once on all emotion recognition tests) emotional recognition ability (without depressive symptoms). Two samples *T*-test corrected by age and sex was used for the statistical test, and the whole brain results were corrected by TFCE (Threshold Free Cluster Enhancement) of permutation test with the threshold of 0.05 (35, 36).

(4) Functional connectivity matrix construction: the resting fMRI data was divided into 250 brain regions according to the Shen template. Nodal signals were created by averaging the regional blood oxygen level-dependent signals of all voxels within each region. Pearson cross-correlations between all pairwise combinations of region signals were calculated to construct the FC correlation coefficient matrix for each participant, followed by Fisher’ Z transformation to make the matrix approximate Gaussian distribution, and each participant constructs an FC matrix.

(5) Mediation analysis was used to examine whether FC strengths mediated the interaction between depressive symptoms and emotion recognition ability after controlling general demographic data.

(6) CONN was used to analyze task-related fMRI data with and without depressive symptoms and for participants with low and high emotional recognition ability.

## 3. Results

### 3.1. Demographic data of the participants

After deleting individuals with missing variables, 995 participants with behavior and brain structure data from HCP were used for the analysis. The participants were young adults, 22–37 years old (mean = 28.76 years, standard deviation = 3.72 years), 464 were male and 75% were white (Table 1). Of 995 participants, 230 had experienced one of the 9 depression diagnostic items in the DSM- IV, and these participants were counted as participants with depressive symptoms.

**TABLE 1:** Demographic and phenotype data characteristics (*n* = 995).

Variables	Mean ± SD/(%)
**Gender**	
Male	464 (46.63%)
Female	531 (53.37%)
Age (years)	28.76 ± 3.72
**Race**	
Caucasian	750 (75.38%)
Other races	245 (24.62%)
Annual household income (dollars)	
<30000	274 (27.54%)
30000–74999	424 (42.61%)
≥75000	297 (29.85%)
Years of education (years)	14.96 ± 1.76
**Left and right handedness**	
Left-handed	68 (6.84%)
Two-handed balance	46 (4.62%)
Right-handed	881 (88.54%)
BMI	26.60 ± 5.23
Urine test positive for addictive drugs	118 (11.85%)
**DSM-4 diagnostic history of substance addiction**	
Marijuana addiction	80 (8.04%)
Alcohol addiction	47 (4.72%)
Tobacco addiction	162 (16.28%)
ASR score of depression syndrome scale	5.93 ± 4.16
Age and sex correction score of ASR depression Syndrome scale	54.03 ± 8.67
ASR depression problems DSM-4 scale score	4.25 ± 2.72
Age and sex correction score of ASR depression Problems DSM-4 scale score	54.05 ± 8.48
ASR anxiety problems DSM-4 scale score	3.94 ± 2.15
Age and sex correction score of ASR anxiety Problems DSM-4 scale score	53.38 ± 8.91
The accuracy of the emotion recognition task	87.52 ± 9.87
The completion time of the emotion recognition task	756.49 ± 91.26

Some of the results are expressed in the form of mean ± SD, some in the form of numerical value and percentage, BMI, body mass index; DSM-IV, fourth edition of Diagnostic and Statistical Manual of Mental Disorders.

### 3.2. Emotion recognition ability was significantly related to depressive symptoms

According to the results of Pearson correlation analysis, it was suggested that there is an extensive pairwise correlation between the accuracy of emotional task completion and the indexes of depressive symptoms (Table 2). Among them, emotion recognition ability was negatively correlated with depressive symptoms (Table 2). These indexes of emotion recognition tasks contained the accuracy percentage of the completed emotion recognition task, the average accuracy percentage of the face recognition task in the emotion recognition task, and the average accuracy percentage of the face shape recognition task in the emotion recognition task. The indexes of depression scale score contained depression syndrome scale score, age and sex corrected depression syndrome scale score, depression DSM score, age and sex corrected depression DSM score. There were positive correlations between the completion time of the emotion recognition tasks and the indexes of depression scale scores.

**TABLE 2:** Correlation analysis and mediating analysis of common brain structure between emotion recognition ability and depressive symptoms.

A/B	The average accuracy of task	Mediating^#^	The average RT of each test during task	Mediating^#^
ASR score of depression syndrome scale	−0.411***	A→B: −0.0021 B→A: −0.0024	0.152*	A→B: 0.002*
				B→A: 0.0023*
Age and sex corrected score of ASR depression syndrome scale	−0.383**	A→B: −0.0062** B→A: −0.0055*	0.133*	A→B: 0.0032**
				B→A: 0.0026*
ASR depression problems DSM-4 scale score	−0.347**	A→B: −0.0059* B→A: 0.0048*	0.129**	A→B: 0.0024
				B→A: 0.0028
Age and sex corrected score of ASR depression problems DSM-4 scale score	−0.328***	A→B: −0.0024* B→A: 0.0021*	0.106*	A→B: 0.0039*
				B→A: 0.0035*

*p < 0.05, **p < 0.01, ***p < 0.001.

^#^Represents mediating analysis of inferior frontal gyrus orbital part between emotion recognition ability and depressive symptoms.

### 3.3. MRI results

#### 3.3.1. Overlapped brain regions related to emotion recognition ability and depressive symptoms

The emotional recognition ability was positively correlated with the cortical thickness of five brain regions, including the right lingual gyrus, the left lingual gyrus, and the operculum part of the inferior frontal gyrus. At the same time, the cortical thickness of three brain regions of the anterior coracoid cingulate cortex, caudate anterior cingulate gyrus, and inferior frontal gyrus orbital part negatively correlated with emotional recognition ability. Among them, the evaluation index of emotion recognition ability was the average reaction time of each test during the completion of the emotion recognition task. The higher the value, the lower the emotion recognition ability. The depressive symptoms were negatively correlated with the cortical thickness of three brain regions, including inferior frontal gyrus orbital part, parahippocampal gyrus and orbitofrontal lobe. Therefore, the cortical thickness of the inferior frontal gyrus orbital part was correlated with depressive symptoms and emotional recognition ability (Table 3).

**TABLE 3:** Brain structures associated with depressive symptoms and emotional recognition ability.

	L/R	β	*p*	*pval_fdr*
**Brain structures associated with depressive symptoms**				
Inferior frontal gyrus orbital part	L	3.029	0.0018	0.037
Parahippocampal gyrus	L	1.599	0.0024	0.046
Orbitofrontal lobe	L	3.187	0.0048	0.047
**Brain structures associated with emotional processing ability**				
Lingual gyrus	R	−72.083	0.0024	0.035
Lingual gyrus	L	−80.524	0.0034	0.044
Tegmental part of the inferior frontal gyrus	L	−79.301	0.0026	0.046
Cuneiform gyrus	R	−66.813	0.0038	0.047
Anterior coracoid cingulate gyrus	L	8.532	0.0018	0.042
Caudate anterior cingulate gyrus	L	15.614	0.0051	0.034
Paratalar gyrus	R	−64.363	0.0029	0.048
Inferior frontal gyrus orbital part	L	14.185	0.0031	0.043

L/R, left/right; β, regression coefficient; *pval_fdr*, *P*-value after FDR correction; *N* = 995.

**FIGURE 1:**
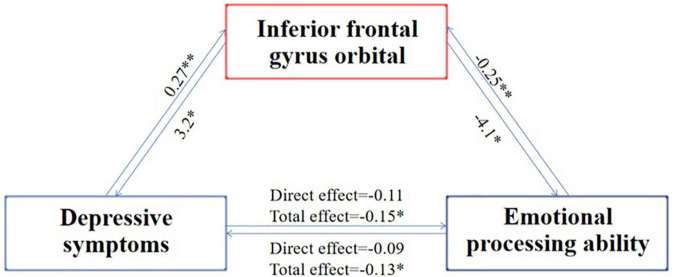
Mediating process of the common brain structure between emotion recognition ability and depressive symptoms. *N* = 995; the index of emotion recognition ability is the average reaction time of each test during the completion of the emotion recognition task, and the index of depression symptom is the score on the depression syndrome scale. The number on the arrow indicates the mediating effect coefficient, ^*^indicates that the *p*-value is less than 0.05, and ^**^indicates that the *p*-value is less than 0.01. The picture shows that the aggravation of depressive symptoms increases the thickness of the cortex of the inferior frontal gyrus orbital part, which in turn deteriorates the ability for emotional recognition, which in turn further increases the thickness of the cortex of the inferior frontal gyrus orbital part, which in turn aggravates the symptoms of depression.

Furthermore, we used simple intermediary analysis to explore whether brain structure mediates the effect of depressive symptoms on emotional recognition ability. The mediating effect and proportion were tested by juxtaposing multiple intermediary analyses when controlling other mediating factors. Table 2 showed a mediating effect between the cortical thickness of the inferior frontal gyrus orbital part, four indexes of depressive symptoms, and two indexes of emotional recognition ability. It could be concluded that depressive symptoms may reduce the emotional recognition ability by affecting the thickness of the inferior frontal gyrus orbital part cortex. In contrast, the decreased emotional recognition ability might also by affecting the thickness of the inferior frontal gyrus orbital part cortex worsen the experience in life and aggravate the depressive symptoms (37–39) (Figure 1).

#### 3.3.2. Functional brain characteristics related to emotional recognition ability and depressive symptoms

**FIGURE 2:**
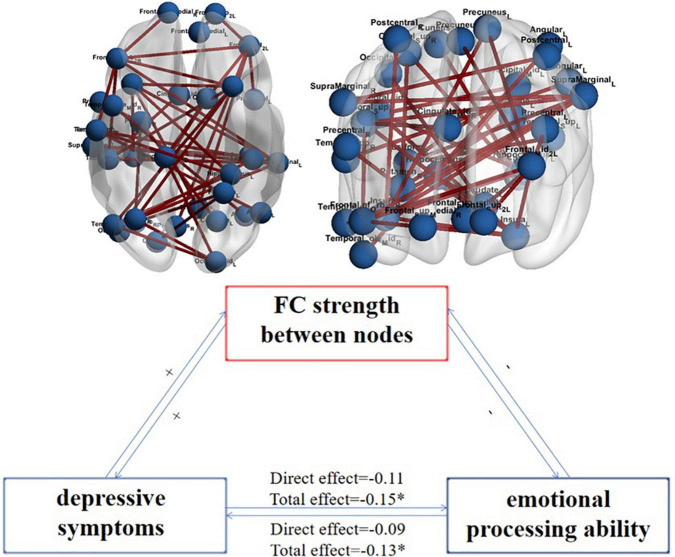
The FC strength between nodes plays an intermediary role in the interaction between emotion recognition ability and depressive symptoms and their schematic representation. *N* = 995; the upper figure shows the FCs that play an intermediary role in the interaction between emotion recognition ability and depressive symptoms; the variables on both sides are the DSM score on the depression scale and the average accuracy of the completed emotion recognition task. The lower figure shows that the aggravation of depressive symptoms increases the strength of FC between nodes (+), which makes the ability of emotional recognition worse (-), making the strength of FC between nodes further increase (+) and then more severe depressive symptoms (-). *Represents total effect is significant.

The strength of FC that plays an intermediary role in the interaction between emotion recognition ability and depressive symptoms is the strength that plays an intermediary role in the process of emotional recognition affecting depressive symptoms and in the process of depressive symptoms affecting emotional recognition ability. By analyzing the FC strength playing an intermediary role in the interaction between emotion recognition ability and depressive symptoms, we obtained 43 shared pairs of FCs. These FCs are the overlap FCs which are significantly related to depressive symptoms and emotional processing ability. Then we further explored whether the identified FCs significantly mediated this association between depressive symptoms and emotional processing ability. The specific FC is shown in Figure 2 upper panel. Figure 2 lower panel is a schematic diagram of the mediating role of FC strength in the interaction between emotion recognition and depressive symptoms.

## 4. Discussion

Depressive symptoms affect emotional recognition ability, and emotional recognition ability, in turn, affects depressive symptoms. Based on the international open database HCP, this study investigated the relationship and overlapped neuroimaging mechanisms between depressive symptoms and emotional recognition ability and also explored the mediating effect of the psychological factors on the interactions.

### 4.1. The relationship between emotion recognition ability and depressive symptoms

There is a negative correlation between emotional recognition ability and depressive symptoms. The score of depressive symptoms was not only negatively correlated with the accuracy of the emotion recognition task but also positively correlated with the reaction time of the emotion recognition task. The results of this study relatively clearly resolved the controversy in previous small sample studies on whether depressive symptoms could affect the ability of emotional recognition in images (9, 40).

### 4.2. sMRI mechanism of the relationship between emotion recognition ability and depressive symptoms

This study further explored the neural mechanism of depressive symptoms and decreased emotional recognition ability, and found that both were related to the cortical thickness of the inferior frontal gyrus orbital part. In individuals with higher levels of depressive symptoms, the inferior frontal gyrus orbital part cortex was thicker. In contrast, the thicker cortex had a lower level of emotional recognition than those with a thinner cortex. And then our results showed that the cortical thickness of the inferior frontal gyrus orbital part mediated the interaction between emotion recognition ability and depressive symptoms, which can partially verify the following hypothesis: the cortex close to the orbitofrontal gyrus is more related to emotional recognition under depressive symptoms.

### 4.3. fMRI mechanism of the relationship between emotion recognition ability and depressive symptoms

Our results showed that the participants who had been diagnosed with depressive disorders had stronger spontaneous activity, higher consistency and lower synchrony of spontaneous activity in the orbital part of the inferior frontal gyrus and the lateral orbitofrontal gyrus; Compared with participants with high emotion recognition ability, participants with low emotion recognition ability had stronger spontaneous activity, higher consistency and lower synchrony of spontaneous activity in the orbital part of the inferior frontal gyrus and the lateral orbitofrontal gyrus. And then, resting-state FC strength between the inferior frontal gyrus orbital part, fusiform gyrus, hippocampus, temporal gyrus, insula, precuneus, and cingulate gyrus mediated the interaction between emotion recognition ability and depressive symptoms. At last, the activation of the frontal pole and precuneus associated with the inferior frontal gyrus orbital part in the emotional recognition task of patients with depression was higher than that of individuals without depression, and the activation of the anterior cingulate gyrus, insula and precuneus associated with the inferior frontal gyrus orbital part in emotional recognition tasks in patients with low completion of emotional recognition tasks were higher, and the activation of the posterior cingulate gyrus and angular gyrus associated with the inferior frontal gyrus orbital part in patients with low completion of emotional recognition tasks was lower. We did find some inconsistency of the brain regions that showed abnormal activities across different modalities. We believe that it is normal that the results of the resting state and task state are inconsistent. Their difference reflects the long-term and short-term differences in the process of emotion recognition. They already have some key brain regions to overlap so that it can be explained. Previous studies are not completely consistent, so we do believe it is acceptable.

Through the above results and previous studies (8), we can identify the critical brain regions involved in emotion recognition, including the orbitofrontal cortex, cingulate cortex, and precuneate lobe et. The orbital prefrontal cortex of humans and other primates is a crucial area of emotional recognition, which evaluates the value of stimuli and whether they are beneficial. The orbitofrontal cortex mainly projects to the anterior cingulate cortex, including its inferior commissural area, then depression is also associated with the anterior cingulate cortex (41). An rs-fMRI FC study of 654 participants shows the lateral orbitofrontal cortex has FC with inferior frontal gyrus (8). The medial orbitofrontal cortex has FC with the para-hippocampal gyrus, hippocampus, temporal cortex, fusiform gyrus, insular lobe, and cingulate gyrus. These FCs play important roles in depressive symptoms and low emotional recognition ability. A similar conclusion is obtained in our results on FC strength. In humans, activation of the medial orbitofrontal cortex is linearly correlated with the subjective (conscious) pleasure of stimulation (42, 43). These reward-related effects were found in pleasant touch, and monetary reward. A recent study of 1140 participants highlighted these ideas, showing that rewards (such as winning a prize or candy) activate the medial orbitofrontal cortex, while not winning activates the lateral orbitofrontal cortex (21). In addition, people with impaired orbitofrontal cortex may be less sensitive to rewards, which is reflected in the decrease in subjective emotion (44). It is difficult for them to recognize facial expressions and emotions related to sound, which is important for emotional and social behavior. It may be the reason for their difficulty in reversing the value of the reward (44). This is consistent with the view found in this study that the cortex near the orbitofrontal cortex is critical in the emotional recognition of patients with depression.

The previous studies and hypotheses on the orbitofrontal lobe (45, 46) were also verified in our research. Our results expand the role of the medial orbitofrontal lobe and lateral orbitofrontal cortex in depressive symptoms through intermediary analysis. The medial and lateral orbitofrontal cortex were distinguished in REHO numerical analysis. In depression, increased cerebral blood flow in areas including the lateral orbitofrontal cortex and cingulate cortex seems to be related to emotional changes because they become more normal when emotional states are relieved (47). The previous studies and hypotheses of FC in brain regions related to depressive symptoms were also verified in our study (20, 23). Our FC results found that the core brain regions such as the inferior orbitofrontal cortex, middle temporal gyrus, precuneate lobe, and cingulate gyrus are the potential neuroimaging basis for the interaction between depressive symptoms and emotional recognition ability.

### 4.4. The limitation of this study

This study has the following limitations: (1) the analysis data are from the international open database HCP, which currently has cross-sectional data, but no longitudinal follow-up data, so it is impossible to explain the long-term trend and causal relationship between emotion recognition ability and depressive symptoms. In the future, longitudinal follow-up studies should be carried out to explore the neural mechanism of emotional recognition ability and depressive symptoms. (2) The imaging data analysis of this study is mainly based on the data-driven method, but there is no preset hypothesis. Both MRI data and fMRI data are analyzed one by one, so the neuroimaging results are scattered, and in-depth data analysis and mining are needed. (3) Depressive symptoms are defined as the presence of one item in nine DSM4 entries, which is different from the entry criteria for the diagnosis of depression. The number of participants also limits this, so an extensive sample database for long-term follow-up is needed.

## 5. Conclusion

This study explores the relationship between emotion recognition ability and depressive symptoms and its neural mechanism based on the young population connectome plan of the HCP sub-project. This study not only elucidates the interaction between the emotional recognition ability and depressive symptoms mediated by the thickness of the inferior frontal gyrus orbital part but also shows that the FC strength of the rs-fMRI of the brain area centered on the inferior frontal gyrus orbital part, such as fusiform gyrus, insular, cingulate gyrus, middle temporal gyrus, precuneate lobe, and hippocampus mediates the interaction between emotion recognition and depressive symptoms. In addition, the predictive analysis of the brain regions and FC of the differences proved that the differences found were predictive.

## Data availability statement

Publicly available datasets were analyzed in this study. This data can be found here: http://www.humanconnectomeproject.org.

## Ethics statement

Written informed consent was obtained from the individual(s) for the publication of any potentially identifiable images or data included in this article.

## Author contributions

All authors listed have made a substantial, direct, and intellectual contribution to the work, and approved it for publication.
