# Supplementary material for: The neural correlation of emotion recognition ability and depressive symptoms–evidence from the HCP database
Source: Front Psychiatry. 2023 Jan 25;13:1090369. doi: 10.3389/fpsyt.2022.1090369 (PMC9905428; doi:10.3389/fpsyt.2022.1090369)
Supplement: Supplementary file 1 [file Data_Sheet_1.docx]

Supplementary materials

The neural correlates of emotion recognition ability and depressive symptoms-- Evidence from the HCP database

Ze Yuan^1 2^ †, Xiao Lin^2^ †, Peng Li^2^, Yu-Jun Gao^3^, Kai Yuan^2^, Wei Yan^2^, Yu-Xin Zhang^2,4^, Liu Lin2 ^4^, Xi-Mei Zhu^2^, Yi-Jing Zhang^2^, Yan-Ping Bao^5,6^ ^*^, Su-Hua Chang^2^ ^*^,Le Shi^2 *^,Lin Lu^2,4,7^ ^*^

^1^ Savaid Medical School, University of Chinese Academy of Sciences, Beijing, China.

^2^ Peking University Sixth Hospital, Peking University Institute of Mental Health, NHC Key Laboratory of Mental Health (Peking University), National Clinical Research Center for Mental Disorders (Peking University Sixth Hospital), Chinese Academy of Medical Sciences Research Unit (No.2018RU006), Peking University, Beijing, China.

^3^ Department of Psychiatry, Renmin Hospital of Wuhan University, Wuhan, China.

^4^ Peking-Tsinghua Centre for Life Sciences and PKU-IDG/McGovern Institute for Brain Research, Peking University, Beijing, China.

^5^ National Institute on Drug Dependence and Beijing Key Laboratory of Drug Dependence, Peking University, Beijing, China.

^6^ School of Public Health, Peking University, Beijing, China.

^7^.National Institute on Drug Dependence and Beijing Key Laboratory of Drug Dependence, Peking University, Beijing, China.

†Equal Author Contribution:

Ze Yuan and Xiao Lin contributed equally as the first authors.

*** Correspondence:**Yan-Ping Bao
[baoyp@bjmu.edu.cn](mailto:baoyp@bjmu.edu.cn)

Su-Hua Chang
[changsh@bjmu.edu.cn](mailto:changsh@bjmu.edu.cn)

Le Shi
[leshi@bjmu.edu.cn](mailto:changsh@bjmu.edu.cn)

Lin Lu
[linlu@bjmu.edu.cn](mailto:linlu@bjmu.edu.cn)

**Supplement Figure 1** Using emotional recognition ability with not including and including inferior frontal gyrus orbital FCs strength predict depressive symptoms respectively

**
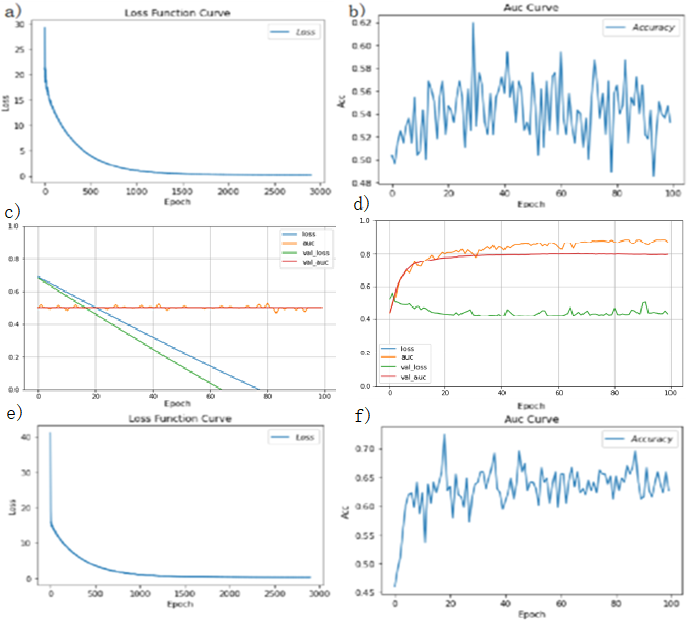
**Legend: N=995; The index of emotional recognition ability selected is the average reaction time of each test during the completion of the emotion recognition task, and the index of depressive symptoms is whether there have been diagnosed depressive symptoms in the past. The a and b pictures show the emotional recognition ability and the strength of the FC that does not include the inferior frontal gyrus orbital part to predict whether there are diagnosed depressive symptoms or not. With the increase of training, the value of the loss function gradually decreases. However, the AUC of artificial neural network prediction still fluctuates around 0.54. It does not improve significantly, indicating that the data can not explore more useful information and is in a relatively randomized state of judgment. On the other hand, the e and f pictures show the emotional recognition ability and the FC strength, including the inferior frontal gyrus orbital part, to predict whether the symptoms of depression have been diagnosed or not. With the increase of the number of training, the value of loss function gradually decreases, and the AUC of artificial neural network prediction fluctuates around 0.64, which is no longer significantly improved, but significantly higher than 0.54 in the above figure. It shows that exploring the FC strength, including the inferior frontal gyrus orbital part, can obtain more effective information than exploring the FC strength that does not include the inferior frontal orbital part, which helps judge whether there are depression symptoms. Figure c shows that the emotional recognition ability is directly used to predict depressive symptoms. With the increase in the number of training, the value of the loss function decreases gradually, but it isn't easy to increase the prediction accuracy. The AUC fluctuates around 0.52. Figure d shows an optimized artificial neural network using the emotional recognition ability and the FC strength, including the inferior frontal gyrus orbital part, to predict whether there is a diagnosis of depressive symptoms. With the increase in training times, the loss function value of the validation group gradually becomes smaller, and the area (abbreviated as AUC) under the curve of the receiver operating characteristic curve (ROC curve) predicted by the artificial neural network to about 0.79. The prediction accuracy has been improved compared with the previous artificial neural network.

**Supplement Figure 2** Using depressive symptoms with not including and including inferior frontal gyrus orbital FCs strength predict Emotional recognition ability respectively


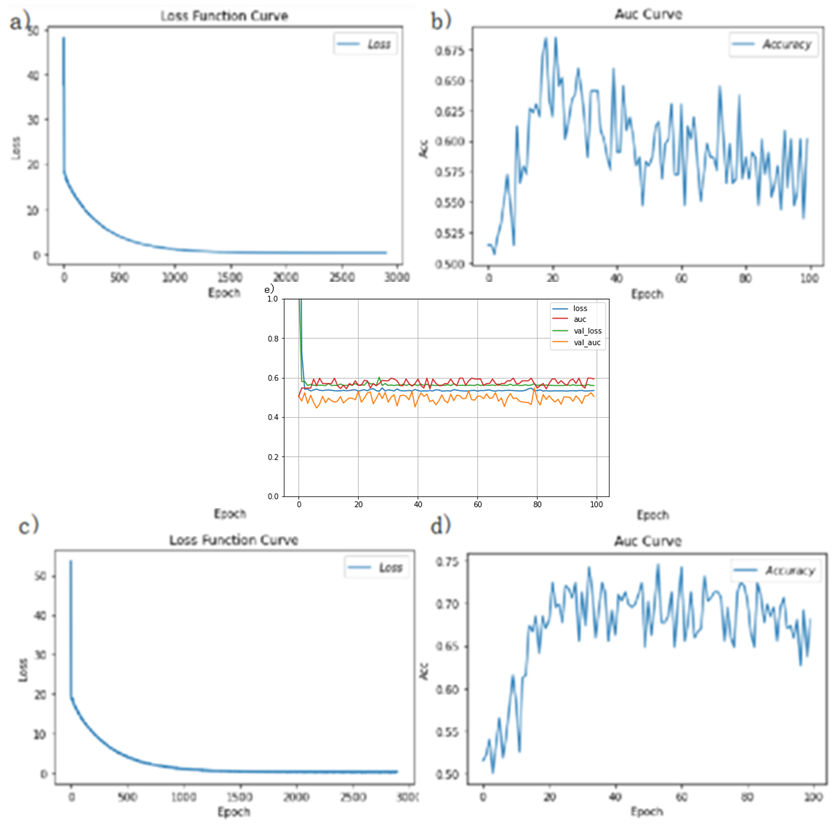


Legend: N=995; The index of emotional recognition ability selected is the average accuracy of the completed emotion recognition task, and the index of depressive symptoms is whether there have been diagnosed depressive symptoms in the past. The a and b pictures show whether there have been diagnosed depressive symptoms in the past and the strength of the FC that does not include the inferior frontal gyrus orbital part to predict emotional recognition ability. With the increase of the number of training, the value of the loss function gradually decreases. However, the AUC of artificial neural network prediction still fluctuates around 0.57. It does not improve significantly, indicating that the data can not explore more useful information and is in a relatively randomized state of judgment. On the other hand, the c and d pictures show using whether there have been diagnosed depressive symptoms in the past and the FC strength, including the inferior frontal gyrus orbital part, to predict emotional recognition ability. With the increase of training, the value of loss function gradually decreases, and the AUC of artificial neural network prediction fluctuates around 0.69, which is no longer significantly improved, but significantly higher than 0.57 in the above figure. It shows that exploring the FC strength, including the inferior frontal gyrus orbital part, can obtain more effective information than exploring the FC strength, which does not include the inferior frontal gyrus orbital part, which helps judge emotional recognition ability. Figure e shows that depressive symptoms are directly used to predict emotional recognition ability. With the increase in the number of training, the value of the loss function decreases gradually, but it isn't easy to increase the prediction accuracy. The AUC fluctuates around 0.53.

**Supplement Figure 3** Differential brain regions of subjects with high and low emotional processing ability by task-fMRI analysis


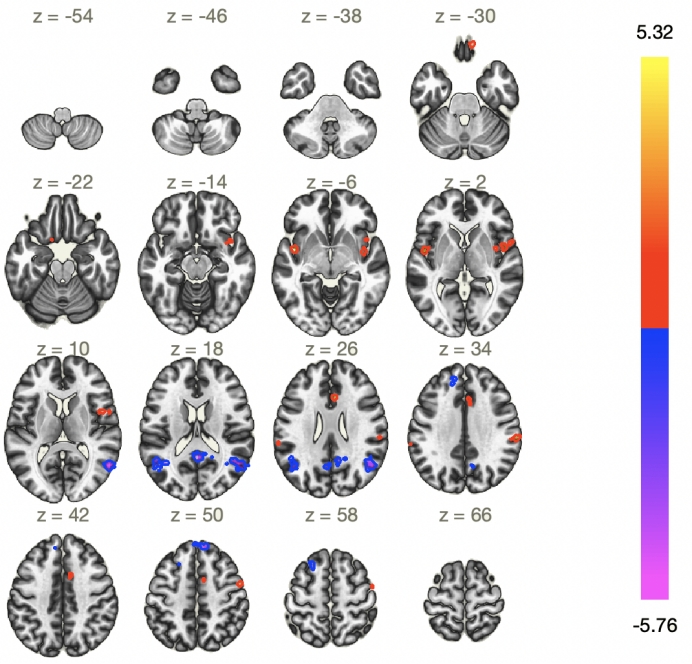


Legend: N=995; In emotional processing tasks, the activation of the anterior cingulate gyrus, insula, and precuneus in emotional processing tasks in patients with low completion of emotional processing tasks are higher than that in patients with high completion, and the activation of the posterior cingulate gyrus and angular gyrus in patients with low completion of emotional processing tasks is lower than that in patients with high completion.

**Supplement Figure** 4 Differential reactivated brain regions in the emotional recognition task in subjects with and without depressive symptoms.


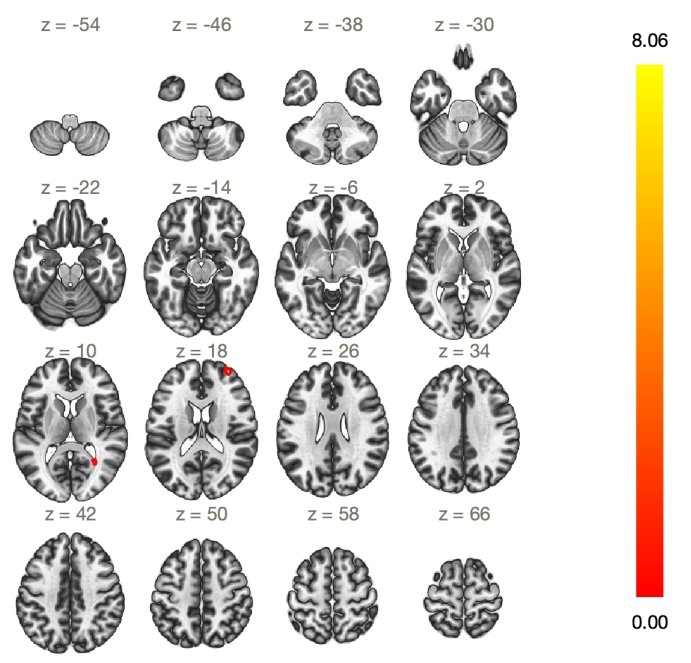


Legend: N=995; the activation of the frontal pole and precuneus in emotional processing tasks in patients with depression is higher than in patients without depression.

**Supplement Figure 5** Whole-brain comparison of ALFF, fALFF, REHO, VMHC values with and without depressive symptoms and participants with low and high emotional recognition ability


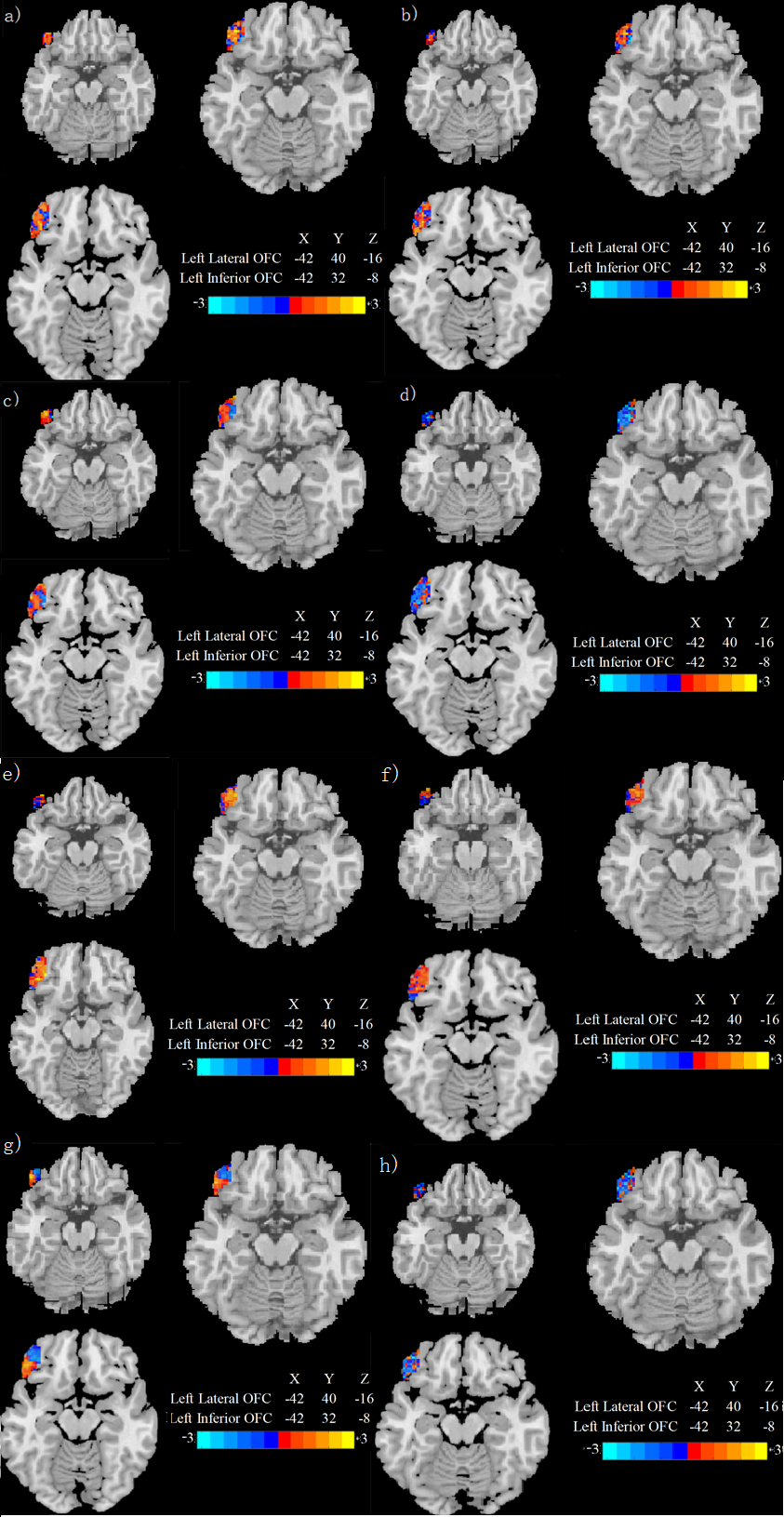


Legend: T-test and TFCE correction of permutation test results showed that comparing participants who have been diagnosed with depressive symptoms in the past with those who have not and comparing participants with low and high emotional recognition ability, the red part means that the former is higher than the latter, while the blue part means that the former is lower than the latter, and the color degree means the degree of TFCE correction result, so the former had higher ALFF, fALFF, REHO and lower VMHC values of most voxels in the inferior frontal gyrus and lateral orbitofrontal gyrus, and the spontaneous activity of the former was stronger, consistency of neural activity of former in the region was higher, and the synchronization of the left and right brain spontaneous activity of former in the region was lower. The displayed brain regions are the parts of the brain regions that showed significant differences in the four resting-state indicators (ALFF, fALFF, REHO, VMHV), that is, the overlapping parts with significant differences in these resting-state indicators. The figure only presented the overlapped brain regions; N=995; figures a e, b f, c g, d h are ALFF, fALFF, REHO, and VMHC, respectively, z=-19, -16, and -14.

**Supplementary Code** Using FCs predicts participants with or without depression symptoms.

import tensorflow as tf
from matplotlib import pyplot as plt
import numpy as np
import pandas as pd
df= pd.read_csv(' .csv') # data
x_data=np.array(df[[ ]]) # X
y_data=np.array(df[[ ]]) # Y
x_train3 = np.vstack(x_data).reshape(-1,x_data.shape[1])
y_train3 = np.vstack(y_data).reshape(-1,1)
x_train = tf.cast(x_train3, tf.float32)
y_train = tf.cast(y_train3, tf.float32)
train_db = tf.data.Dataset.from_tensor_slices((x_train, y_train)).batch(32)
w1 = tf.Variable(tf.random.normal([x_data.shape[1], 32]), dtype=tf.float32)
b1 = tf.Variable(tf.constant(0.01, shape=[32]))
w2 = tf.Variable(tf.random.normal([32, 1]), dtype=tf.float32)
b2 = tf.Variable(tf.constant(0.01, shape=[1]))
 lr = 0.01
epoch = 100
train_loss_results = []
prob = []
acc_all= []
y2=0
for epoch in range(epoch):
 for step, (x_train, y_train) in enumerate(train_db):
 with tf.GradientTape() as tape:
 h1 = tf.matmul(x_train, w1) + b1
 h1 = tf.nn.relu(h1)
 y = tf.matmul(h1, w2) + b2
 loss_mse = tf.reduce_mean(tf.square(y_train - y))
 loss_regularization = [] loss_regularization.append(tf.nn.l2_loss(w1))
 loss_regularization.append(tf.nn.l2_loss(w2))
 loss_regularization=tf.reduce_sum(loss_regularization)
 loss = loss_mse + 0.15 * loss_regularization
 train_loss_results.append(loss)
 variables = [w1, b1, w2, b2]
 grads = tape.gradient(loss, variables)
 w1.assign_sub(lr * grads[0])
 b1.assign_sub(lr * grads[1])
 w2.assign_sub(lr * grads[2])
 b2.assign_sub(lr * grads[3])
 if epoch % 20 == 0:
 print('epoch:', epoch, 'loss:', float(loss))
 X_train,X_test,y_train2,y_test = train_test_split(x_train3,y_train3,test_size =0.3)
 grid = to.cast(X_test,tf.float32)
 probs = []
 w=0
 total_correct, total_number = 0, 0

 for x_test in grid:
 h1 = tf.matmul([x_test], w1) + b1
 h1 = tf.nn.relu(h1)
 y = tf.matmul(h1, w2) + b2
 if y>0.5:
 y=1
 if y<0.5:
 y=0
 probs.append(y)
for i in range(len(probs)):
 if probs[i]==y_test[i]:
 w=w+1
 acc=w/len(probs)
 acc_all.append(acc)
 if epoch % 20 == 0:
 print("Test_acc:", acc)
plt.title('Loss Function Curve')
plt.xlabel('Epoch')
plt.ylabel('Loss')
plt.plot(train_loss_results, label="$Loss$")
plt.legend()
plt.show()
plt.title('Acc Curve')
plt.xlabel('Epoch')
plt.ylabel('Acc')
plt.plot(acc_all, label="$Accuracy$")
plt.legend()
plt.show()

**Supplementary process of prediction**

We also established an artificial neural network (code as shown above) to predict depressive symptoms and emotional recognition ability. We also tried to make a prediction using the rs-fmri function indicators (alff,falff,reho,vmhc) and T1 images. Still, the prediction efficiency was poor, mainly because the regions with significant differences in rs-fmri functional indicators were scattered and unintegrated. The prediction accuracy of a single rs-fmri indicator was only about 55%, and the accuracy of the combined T1 image and four rs-fmri indicators was also about 60%, which was weak. Then we analyzed the task-state fMRI of the emotional recognition task. We tried to make prediction using task-state fMRI because the brain regions with significant differences in tasking state are small, and the scanning time is short; the effect of prediction analysis is poor, less than 60%, so it is not shown. It was proved that the 43 pairs of FC strengths centered on the orbital part of the inferior frontal gyrus had specific predictive power.

**Supplementary process of UKB validation**

We extracted data from the UKB database of 326 participants who had been diagnosed with depression and had not been diagnosed with other neurological or psychiatric disorders and had undergone rs-fmri and T1 MRI scanning and 400 healthy participants who had not been diagnosed with neurological or psychiatric disorders and had undergone rs-fmri and T1 MRI scanning and performed FC calculation with DPABI. The HCP database-trained artificial neural network was used to distinguish between diagnosed and undiagnosed depressed participants in the UKB database. Finally, compared with the actual label, it was found that the prediction accuracy was 70.9%. The model can identify patients with depression with an accuracy of up to 70%.
